# Subjectively Reported Symptoms in Patients with Persistent Atrial Fibrillation and Emotional Distress

**DOI:** 10.3389/fpsyg.2013.00192

**Published:** 2013-04-24

**Authors:** Nina Kupper, Krista C. van den Broek, Jos Widdershoven, Johan Denollet

**Affiliations:** ^1^Center of Research on Psychology in Somatic diseases, Tilburg UniversityTilburg, Netherlands; ^2^Department of Cardiology, TweeSteden hospitalTilburg, Netherlands

**Keywords:** atrial fibrillation, depression, anxiety, subjectively reported AF symptoms, cardioversion

## Abstract

**Background:** Patients with atrial fibrillation (AF) are characterized by emotional distress and poor quality of life. Little is known about the relation between emotional distress and subjectively reported AF symptoms. Our aims were to compare emotional distress levels in AF patients with distress levels in the general population and to examine the cross-sectional and prospective relationship between subjective AF symptom reports and emotional distress around electrical cardioversion (ECV).

**Methods:** At baseline, this study included 118 patients with persistent AF planned for ECV (aged 68 ± 10 years, 60% men) in which depression (BDI), anxiety (STAI), Type D personality (DS14), perceived stress (PSS-10), and AF symptoms (ATSSS) were assessed. The prospective substudy included 52 patients. Objective AF status was determined by ECG.

**Results:** AF patients experienced significantly higher levels of anxiety (*p* < 0.001) and depression (*p* < 0.001) than age and gender matched persons from the general population. Linear regression analyses showed that AF patients with higher depression levels reported significantly more AF symptoms (β = 0.44; *p* < 0.0005) and reported symptoms to occur with a higher frequency (β = 0.51; *p* < 0.0005) during the AF episode, independent of age, sex, cardiac disease, BMI, and physical activity. At 4 weeks follow-up, 56% of all patients had maintained sinus rhythm. Repeated Measures Linear mixed modeling showed that these patients reported fewer AF symptoms and a lower frequency of AF symptoms pre and post-ECV (*p* = 0.04). Also, the course of the number and frequency of reported symptoms was significantly associated with the change in depression over that same time period (*p* < 0.0005).

**Conclusion:** Patients with persistent AF are characterized by emotional distress. Distressed AF patients, particularly the depressed, report more AF symptoms before and after ECV. These findings call for increased attention of clinicians to emotional distress in this patient population.

## Introduction

Atrial fibrillation (AF) is the most common, multi-etiologic cardiac arrhythmia characterized by disorganized electrical signaling in and from the cardiac atria, which results in an increased and irregular heart beat. AF by itself is not life-threatening, but it does increase the risk of embolic complications, stroke, cognitive impairment, and congestive heart failure (Camm et al., 2009).

Atrial fibrillation is generally accompanied by a poor quality of life (QoL) that is substantially lower than in age and gender matched persons from the general population (Hagens et al., 2004; Lane et al., 2009). Reduced QoL may be related to common AF symptoms such as fatigue and shortness of breath (Kang, 2006b). Other QoL reducing factors may include the chronic character of the disease, accompanied by chronic use of medication, medication side effects, the underlying cause of AF, as well as the frequently occurring rhythm control interventions, such as electrical cardioversion (ECV) (Thrall et al., 2006), although symptom alleviation does improve QoL (Smith et al., 2010).

Studies in cardiac patients, including AF patients, have shown that emotional distress is related to impaired QoL (Lane et al., 2001; van den Berg et al., 2005; Ong et al., 2006). Further investigation of emotional distress in AF patients is generally lacking. Studies have indicated that anxiety may be more prevalent in AF patients than depression (Lane et al., 2009), and that both anxiety and depression are rather persistent, with depression being an important predictor of future QoL (Thrall et al., 2007). The effects of emotional distress on disease prognosis are yet unclear, with one study indicating that depression may be related to AF recurrence after successful ECV, whereas anxiety and the distressed personality may not (Lange and Herrmann-Lingen, 2007). On the other hand, a recent study demonstrated that Type D personality was more prevalent in patients with an emotionally triggered stress cardiomyopathy (76% Type D) compared to stress cardiomyopathy patients without an emotional trigger (43% Type D) or patients with myocardial infarction with an emotional trigger (32% Type D), carefully suggesting that Type D personality may contribute to the risk of emotionally triggered cardiomyopathy (Compare et al., 2013).

There is also limited knowledge on the association between emotional distress and symptomatic burden. Recently, an argument was made to include subjective symptomatic burden as an alternative endpoint in clinical AF trials (Darbar and Roden, 2005), since the increased short-term risk of mortality and events such as stroke in AF patients is small and therefore long follow-up periods are needed. These hard endpoints do not measure symptoms, which is what the patient experiences (Darbar and Roden, 2005), and which are in turn related to AF recurrence (Darbar and Roden, 2005). In addition, the goal of AF treatment is often to reduce symptom burden. Patients with higher levels of emotional distress may report more AF symptoms. One study in patients with newly diagnosed AF and one in a convenience sample of AF patients with concomitant cardiovascular disease or lone AF showed that emotional distress was positively associated with the reported severity of AF symptoms (Kang, 2006a; Ong et al., 2006). In addition, Sears and colleagues showed that the number of reported AF symptoms were mostly influenced by the distress state of patients, and to a lesser extent by objective indicators of AF (Sears et al., 2005). However, anxiety and depression were transformed into one “negative emotions” variable in this study, potentially precluding differential effects of anxiety and depression on symptom reports. In addition, a call was made for additional studies to be carried out as the abovementioned study used a highly selected sample of AF patients fitted with an implantable cardioverter defibrillator with atrial therapies.

Therefore, the objectives of the current study were (1) to determine the prevalence of emotional distress, including anxiety, depression, distressed (Type D) personality, and perceived stress, in patients with persistent AF during an AF episode, and to compare these emotional distress levels with levels in an age and gender matched sample from the general population, (2) to examine the association between emotional distress and subjective report of AF symptoms during an AF episode, and (3) to study the effects of emotional distress on the changes in subjective symptom reports from before to 4 weeks after ECV.

## Materials and Methods

### Patient population

Between October 19, 2007 and March 4, 2009 a total of 205 patients diagnosed with persistent AF and eligible for ECV presented at the Cardiology clinic of the TweeSteden hospital for an elective ECV to restore their sinus rhythm (SR). Of these, 124 agreed to participate in the current survey study by returning the questionnaire before cardioversion. Patients who declined to participate did not differ from participants of the study with regard to gender (*p* = 0.52) or age (*p* = 0.50). Patients (*n* = 6) with AF due to cardiac surgery or valvular dysfunction were excluded from analysis (final sample *n* = 118). A substudy examined the course of AF symptoms from before to 6 weeks after the cardioversion. Fifty-two patients participated in this substudy. AF status at inclusion and follow-up were determined by ECG. The study protocol was approved by the hospital’s local medical ethics committee in Tilburg, Netherlands (METOPP) as this behavioral study was not subject to the Medical Research Involving Human Subjects Act (WMO). The study was conducted conform to the most recent version of the Helsinki Declaration (2008) and all patients provided written informed consent prior to participation.

### General population

We matched the AF patients to healthy control subjects with respect to gender and age. The healthy controls were drawn from a random selection of 2419 adults from the general Dutch population that had participated earlier in a large survey study on psychological and physical health, and for which data on Type D personality (DS14), state anxiety (STAI), and depression (BDI) were present. We used participants’ self-report on medical diagnoses (e.g., “Do you have high blood pressure?” “Do you have a respiratory disease?”) to establish that they were healthy. Information on how these data were collected has been published previously (Mols et al., 2009). All participants signed written informed consent and the university ethics committee approved the study protocol.

### Socio-demographic and clinical variables

Socio-demographic information was obtained via self-report and included gender, age, education, and marital status. Smoking status, alcohol use, physical activity status, and anthropometric characteristics were also assessed by means of self-report. Clinical variables were obtained from medical records and included co-morbid (previous thromboembolism, stroke, transient ischemic accident, cancer, peripheral arterial disease, and renal insufficiency) and concomitant (hypertension, coronary artery disease, heart failure, valvular heart disease, cardiomyopathy, sick sinus syndrome, COPD, and thyroid disease) diseases, current medication use, previous cardioversions (electrical and pharmacological), and successfulness of the ECV at follow-up.

### Subjective AF symptom report

The Atrial Tachyarrhythmia Symptom Severity Scale (ATSSS) (Maglio et al., 1998; Dorian et al., 2000) is a 16-item questionnaire asking the patient to report which AF symptoms have been experienced over the past 4 weeks, along with the frequency and severity of the experienced AF symptoms. The frequency of symptoms was rated on a 5-point Likert scale from 1 (never) to 5 (all the time). We used two scores: (1) the total number of symptoms reported (range 0–16); (2) total AF symptom frequency score, i.e., the summation of the rated frequency of all 16 items (range 16–80).

### Psychological assessment

Patients completed a psychological survey in the week before the ECV, and at on average 4.5 weeks thereafter (coinciding with the follow-up visit at the clinic). Patients were informed about the study by their attending cardiologist and received an information letter, informed consent form, and the psychological questionnaire in a closed envelope. When willing to participate, patients filled out the questionnaire and brought the questionnaire and the signed consent form with them to the cardioversion appointment. The follow-up questionnaire came with a pre-stamped, self-addressed envelope the patients were asked to use to return the filled out questionnaire.

### Emotional distress

#### Depression

Depressive symptomatology was assessed with the 21-item Beck Depression Inventory (BDI-I) (Beck and Steer, 1993). Each item is rated on a 0–3 Gutmann scale. A total score is obtained by summing together all items. Total scores range from 0 to 63, with scores above 10 indicating at least mild levels of depression (Beck and Steer, 1993). The BDI is a reliable and well-validated measure of depressive symptomatology (Beck et al., 1988), and is a widely used self-report measure of depression in patients with cardiovascular disease.

#### Anxiety

State anxiety was assessed by the 20-item State subscale of the Spielberger State Trait Anxiety Inventory (STAI) (Ploeg van der et al., 1980; Spielberger, 1983). All items are rated on a 4-point Likert scale from 1 (not at all) to 4 (very much so), with a score range of 20–80. Positively stated items (*n* = 10) were reversed before summing. A high score indicates high levels of anxiety, with a score ≥40 indicating probable levels of clinical anxiety (Ploeg van der et al., 1980; Spielberger, 1983). The STAI is a valid and reliable scale, with Cronbach’s α ranging from 0.87 to 0.92 (Ploeg van der et al., 1980).

#### Perceived stress

The Perceived Stress Scale (PSS) is a 10-item questionnaire measuring of the degree to which, over the past month, life situations may be appraised as stressful (Cohen and Williamson, 1988). Answers are given on a 5-point Likert scale from 0 (never) to 4 (very often). PSS scores are obtained by reversing responses to the four positively stated items and then summing across all scale items (Cohen and Williamson, 1988). In the current sample, PSS had good psychometric properties, with Cronbach’s alpha being 0.82.

#### Distressed personality (Type D)

Type D personality reflects the general tendency to experience negative emotions, while at the same time inhibiting these emotions in social situations. Type D personality was assessed by the DS14 (Denollet, 2005), consisting of 14 items that are divided into two subscales measuring two stable personality traits, negative affectivity (NA; tendency to experience negative emotions across time and situations), and social inhibition (SI; tendency to inhibit the expression of negative emotions in social interaction). The items are answered on a 5-point Likert scale ranging from 1 (false) to 4 (true). Scores on the two positively worded items in the SI subscale were reversed before summing. A standardized cut-off ≥10 on both subscales indicates Type D caseness. The two subscales have good psychometric qualities, with Cronbach’s α = 0.88/0.86 and 3 month test-retest reliability *r* = 0.72/0.82 for the negative affectivity and social inhibition subscale, respectively (Denollet, 2005).

### Statistical analysis

Baseline characteristics were calculated using descriptive statistics [means (SD) and frequencies]. Pearson correlations were used to calculate the relation between continuous emotional distress scores and AF symptom number and frequency scores. Point-biserial (Pearson) correlations were used to assess the correlation of the symptoms scores and the dichotomous Type D personality score. Student *t*-tests for independent samples were used to assess differences between the AF patient group and the general population sample and paired *t*-tests were used to examine change in the report of individual AF symptoms before and after ECV, as well as differences in emotional distress levels and subjective symptom reports between patients still in SR at 4 weeks after ECV versus patients that had reverted to AF rhythm.

#### Covariates

Since age and gender related differences have been reported in relation to AF (Rich, 2009; Volgman et al., 2009), and obesity (Lavie et al., 2009) and physical exercise (Rich, 2009) have been associated with AF as well, we included age, gender, BMI, and engagement in physical activity in the multiple regression analyses. We also included concomitant cardiovascular diseases CAD and CHF as covariates, as we also wanted to discriminate between AF patients with and without concomitant cardiovascular disease. In the mixed linear modeling analysis for repeated measures we only included the significant covariates from the cross-sectional regression analysis.

Multiple linear regression analyses examined the influence of the various measures of emotional distress on subjectively reported AF symptoms in the presence of the priori selected covariates.

The mixed linear modeling procedure in SPSS 17.0.3 (SPSS Inc. Chicago, USA) was used to examine the effects of emotional distress on the change in number and frequency of reported AF symptoms before and 4 weeks after ECV while taking into account the successfulness of the ECV and significant covariates from the multiple regression analysis. This technique is suitable for analysis of repeated measurements, as it reckons with the possibility of correlated data. In addition, in contrast to traditional repeated measures analysis of variance, one missing measurement occasion does not automatically lead to exclusion of that patient from analysis, limiting bias and preserving statistical power. All cases are therefore used in the mixed modeling analysis. Data was first restructured by the *variables to cases* procedure such that per patient consecutive AF symptom reports and emotional distress measurements (baseline and follow-up) constituted a case. We further used an unstructured covariance matrix, so that no *a priori* assumptions were made about the relatedness of the follow-up data. Then, we performed two analyses, one for the number of reported AF symptoms and one for the frequency of the reported AF symptoms (dependent variables). Each analysis consisted of three steps. In the first step, we added time and maintenance of SR as factors. Next, we added the significant covariates from the linear regression as constant covariates. Finally, all emotional distress measures (depression, anxiety, and perceived stress) were entered as time-varying continuous factors. All analyses were two-tailed and a *p* value of <0.05 was considered significant.

## Results

### Patient characteristics

Table 1 shows the baseline patient characteristics. The majority of the patients was male (62%) and patients were on average 68 years of age. Approximately half of the sample had concomitant cardiac disease, while the other half had lone AF or AF with hypertension. Sixty percent of patients had a previous ECV or pharmacological intervention before inclusion into the current study. Due to the short follow-up time, at 4 weeks follow-up only half of the sample (*n* = 52) had returned their follow-up questionnaire. *t*-Tests and chi square tests showed that those who participated only once did not differ from those who participated twice on baseline characteristics including age (*t* = −1.5, *p* = 0.13), gender (χ^2^ = 3.6, *p* = 0.06), number (*t* = 0.7, *p* = 0.45), and frequency (*t* = 0.9, *p* = 0.40) of reported symptoms, maintenance of SR at follow-up (χ^2^ = 2.1, *p* = 0.15) and levels of emotional distress (anxiety: *t* = −1.4, *p* = 0.18; depression: *t* = −0.3, *p* = 0.97; perceived stress: *t* = 0.6, *p* = 0.79; Type D personality: χ^2^ = 0.3, *p* = 0.59).

**Table 1:** **Patient characteristics**.

	Persistent AF (*N* = 118)
**DEMOGRAPHICS**	
Age (years) Mean ± SD	68.0 (±9.4)
Female gender	38.8% (38)
Time since diagnosis (years) Mean ± SD	3.8 (±4.6)
**CONCOMITANT DISEASE**	
Hypertension	46.6% (55)
Coronary artery disease	35.6% (42)
Heart failure	12.7% (15)
Valvular heart disease	3.4% (4)
Cardiomyopathy	6.8% (8)
Sick sinus syndrome	1.7% (2)
COPD	7.6% (9)
Thyroid disease	7.6% (9)
Idiopathic disease	44.1% (52)
**CARDIOVASCULAR RISK FACTORS**	
Diabetes	12.7% (15)
Hyperlipidemia	35.9% (42)
Smoking	10.9% (11)
Lack of exercise	54.6% (56)
Family history of CAD	32.1% (36)
**COMORBIDITIES**	
Previous thromboembolism	2.5% (3)
Stroke	3.4% (4)
TIA	3.4% (4)
Cancer	6% (7)
Peripheral arterial disease	5.9% (7)
Renal insufficiency*	26.1% (26)
**PREVIOUS INTERVENTIONS**	
Pharmacological cardioversion	8.4% (10)
Electrical cardioversion	51.8% (60)
Catheter ablation	0%
ICD implantation	3.4% (4)
Surgery for AF	1.7% (2)
**DRUG THERAPY AT STUDY INCLUSION**	
Antithrombotic	
Oral anticoagulation	98% (115)
Anti-arrhythmic/rate control	
Type Ia	8% (9)
Type Ic	8% (9)
Type II	44% (51)
Type III	55% (64)
Type IV	11% (13)
Digoxin	21% (24)
Other	
ACE inhibitor	33% (39)
ARB	17% (20)
Beta-blocker (other than Type II)	3% (3)
Diuretic	30% (35)
Spironolactone	7% (8)
Statin	39% (46)

*Based on glomerular filtration rate of creatinine, using the MDRD formula.

### Subjective AF symptom report

During the AF period, patients reported the presence of on average 9.3 (±4.1) symptoms (out of 16), with a frequency of on average 2.5 (±0.5; range 2–4) for those symptoms that were reported to be present. Table 2 shows the prevalence and the mean frequency for each subjective AF symptom, both at baseline and 4 weeks follow-up. The majority of patients reported symptoms of tiredness, dyspnea and tachycardia, while the least reported symptoms were nausea, poor appetite and chest pain in absence of tachycardia. Paired *t*-tests showed that patients reported significantly less heart racing 4 weeks after cardioversion and reported trend reductions in heart fluttering or beat skipping and accompanying chest pain (Table 2). Of note, there were no gender differences in the number or frequency of reported AF symptoms before or after ECV.

**Table 2:** **Prevalence and mean frequency of the individual subjective AF symptoms**.

AF symptom	Prevalence during AF episode (%)	Mean frequency (SD) during AF episode*	Prevalence at follow-up	Mean frequency (SD) at follow-up
Tiredness/lack of energy	92	3.0 (1.0)	92	2.8 (1.1)
Shortness of breath	76	2.8 (0.9)	71	2.6 (0.9)
Hard to catch breath	75	2.6 (0.9)	67	2.6 (0.9)
Heart racing	70	2.9 (1.0)	48	2.5 (0.9)*
Heart fluttering, skipping	69	2.9 (1.0)	46	2.5 (0.9)^†^
Weakness	63	2.5 (0.8)	52	2.3 (0.6)
Sweating	63	2.5 (0.7)	47	2.3 (0.6)
Trouble concentrating	62	2.4 (0.7)	68	2.3 (0.6)
Light-headedness/dizziness	62	2.4 (0.7)	59	2.3 (0.7)
Difficulty sleeping	58	2.7 (0.8)	65	2.5 (0.7)
Chest pain, pressure or fullness when heart is racing or fluttering	52	2.3 (0.6)	46	2.2 (0.5)^†^
Headache	46	2.4 (0.7)	44	2.7 (1.0)
Feeling warm, flushed	44	2.4 (0.7)	40	2.2 (0.5)
Chest pain, pressure or fullness when heart is not racing or fluttering	32	2.3 (0.7)	35	2.0 (0.0)
Poor appetite	31	2.6 (0.9)	27	2.5 (0.8)
Nausea	24	2.3 (0.7)	27	2.1 (0.3)

**Total scores**		**Mean total**		**Mean total**

Number of reported symptoms		9.0 (4.0)		8.3 (3.4)
Frequency of reported symptoms		30.7 (9.1)		28.4 (8.1)

Frequency of AF symptoms is rated from 1 (never) to 5 (all the time). A symptom is present when a score of 2 or higher is recorded. *Mean frequency was determined for patients that reported the symptom to be present [2 (some of the time) – 5 (all the time)]. Paired *t*-tests were done on the original frequency variables [including 1 (absence of symptom)]; *Significant reduction (*p* < 0.05) ^†^ trend reduction (*p* < 0.10).

### Comparing emotional distress in AF patients with matched healthy controls

Emotional distress levels were relatively high in the patients with persistent AF (see Table 3, left panel). Levels in AF patients were significantly higher in comparison to the matched healthy control group (for depression: 8.6 ± 7.1 versus 5.6 ± 4.5; *t* = −4.22, *p* < 0.001; for anxiety: 37.5 ± 12.1 versus 32.2 ± 7.4; *t* = −3.80, *p* < 0.001). A significantly larger number of AF patients (33%) showed signs of clinically significant depression compared to healthy controls (13%; χ^2^ = 10.72, *p* = 0.001). Similar findings were found for anxiety, as 42% of AF patients reported clinically relevant levels of anxiety compared to 11% of the healthy controls (χ^2^ = 20.85, *p* < 0.001).

**Table 3:** **Prevalence of emotional distress during the AF episode and after sinus rhythm had been restored**.

Emotional distress	During AF episode (*n* = 118)		After ECV			
			Sinus rhythm at 4 weeks FU (*n* = 31)		AF rhythm at 4 weeks FU (*n* = 21)	
	Mean (SD)	%	Mean (± SD)	%	Mean (±SD)	%
Type D personality	–	28	–	–	–	–
Depression	8.6 (6.8)	32.7	8.1 (5.1)	29.0	9.1 (5.3)	38.1
Anxiety	37.5 (12.1)	42	37.7 (9.7)	36.7	38.4 (13.8)	50.0
Perceived stress	15.8 (7.9)		15.3 (6.2)		15.4 (7.9)	

*ECV, electrical cardioversion; AF, atrial fibrillation; FU, follow-up.% Depression,% Anxiety: percentage of patients scoring above a predefined threshold for clinically relevant depression and anxiety*.

Type D personality prevalence was 28% in AF patients, which was to be expected in a cardiac patient population (Kupper and Denollet, 2007). Type D personality prevalence in the healthy controls was lower, with a prevalence of 17% (χ^2^ = 3.48, *p* = 0.06).

### Subjective symptom reports during the AF episode and emotional distress

The correlations presented in Table 4 show that depression, anxiety and perceived stress, but not Type D personality, were highly related to the number as well as the frequency of reported symptoms of AF. Linear regression analyses showed that depression (β_number_ = 0.58, *p* = < 0.001; β_frequency_ = 0.52, *p* < 0.001), anxiety (β_number_ = 0.35, *p* = 0.002; β_frequency_ = 0.28, *p* = 0.008), and perceived stress (β_number_ = 0.41, *p* < 0.001; β_frequency_ = 0.32, *p* = 0.003) were all significantly related to respectively the number and the frequency of reported AF symptoms during the AF episode, independent of the *a priori* selected covariates (Table 5).

**Table 4:** **Relation of emotional distress with the number and frequency of reported AF symptoms during the AF episode and 4 weeks post-ECV**.

	During AF episode (*n* = 118)		4 weeks post-ECV (*n* = 52)			
	Number of AF symptoms	Frequency of AF symptoms	Number of AF symptoms		Frequency of AF symptoms	
			SR	AF	SR	AF
Type D personality	0.08	0.12				
Depression	0.53***	0.55***	0.39*	0.38	0.46*	0.50*
Anxiety	0.30**	0.26**	0.60**	0.36	0.59**	0.40^†^
Perceived stress	0.37***	0.29**	0.39*	0.39^†^	0.36^†^	0.43^†^

*SR, patients still in sinus rhythm 6 weeks post-ECV; AF, patients that relapsed into AF rhythm 4 weeks post-ECV*.

^†^ = trend (p < 0.10), * = p < 0.05, ** = p < 0.01, *** = p < 0.001.

**Table 5:** **Multivariable regression analyses on the effects of emotional distress on the number and severity of reported AF symptoms during the AF episode**.

	During AF episode (*n* = 118)					
	Number of AF symptoms			Frequency of AF symptoms		
	β	*P*	*R*^2^	β	*P*	*R*^2^
**MODEL 1**						
All covariates*			0.11			0.07
CAD	0.22	0.04		**0.25**	**0.03**	
Physical activity	0.18	0.09				
**MODEL 2**						
+*Depression, anxiety &* perceived stress*			0.44			0.32
CAD	0.18	0.051		**0.20**	**0.046**	
**Physical activity**	**0.22**	**0.02**				
**Depression**	**0.45**	**<0.0005**		**0.54**	**<0.0005**	
State anxiety	0.04	0.74		0.005	0.97	
Perceived stress	0.07	0.59		−0.01	0.93	

*Covariates in the model: presence of cardiovascular disease (CAD, CHF), BMI, age, gender, and physical activity. Only significant (or trend) covariates are shown in step 1.

*Bold faced: significant (p < 0.05) relations in the model*.

When entering all measures of emotional distress into one regression analysis, including the *a priori* selected covariates, only depression remained a significant associate of both the number and frequency of reported AF symptoms (Table 5). In this multivariate model, physical activity and CAD were other associates of the number and frequency of AF symptoms, with more and more frequent report of symptoms in patients with CAD and in patients that performed regular physical exercise at least once a week. Multicollinearity diagnostics showed that tolerance was acceptable (>0.47), making multicollinearity improbable and justifying the inclusion of the three emotional distress measures together in one analysis.

### Determinants of the change in number and frequency of subjective symptoms

Four weeks after the ECV, 56% of patients were still in SR.

#### Number of symptoms

Linear mixed modeling of the repeated assessments of the AF symptom reports and the emotional distress questionnaires showed that the baseline and post-ECV symptom reports did not differ significantly from each other, as there was no effect of time (Table 6).

**Table 6:** **Associates of change in the reported number and frequency of AF symptoms before and after ECV**.

	Number of AF symptoms				Frequency of AF symptoms			
	Estimate	SE	*t*	*p*	Estimate	SE	*t*	*p*
**MODEL 1**								
Time	0.33	0.56	0.6	0.56	1.53	0.94	1.6	0.11
AF status at FU	−1.7	0.81	−2.1	**0.04**	−3.7	1.81	−2.1	**0.04**
**MODEL 2**								
Time	0.31	0.60	0.5	0.61	1.76	1.01	1.7	0.09^†^
AF status at FU	−1.7	0.83	−2.1	**0.04**	−3.8	1.95	−1.9	0.056^†^
Presence of CAD	−1.0	0.77	−1.3	0.19	−3.2	1.8	−1.7	0.09^†^
Physical activity	−2.0	0.77	−2.5	**0.01**	−1.5	1.84	1	0.41
**MODEL 3**								
Time	0.03	0.62.	0.1	0.96	1.37	1.18	1.2	0.25
AF status at FU	−1.3	0.88	−1.5	0.15	−2.43	1.88	−1.3	0.20
Presence of CAD	−0.7	0.81	−0.9	0.38	−1.12	1.71	−0.7	0.52
Physical activity	−2.5	0.83	−3.0	**0.004**	−3.48	1.8	−1.9	0.06^†^
Depression	0.30	0.08	3.9	**<0.0005**	0.74	0.16	4.5	**<0.0005**
Anxiety	0.03	0.04	0.7	0.46	0.07	0.09	0.8	0.43
Perceived stress	−0.03	0.07	−0.5	0.62	−0.13	0.14	−0.9	0.36

*SE, standard error; FU, follow-up; CAD, coronary artery disease; boldfaced, *p* < 0.05; ^†^, trend (*p* < 0.10). Estimate can be interpreted as a unstandardized linear regression coefficient, meaning that 1 point increase in, e.g., depression from baseline to follow-up will lead to an [estimate] increase/decrease in the number/frequency of AF symptoms over that time period*.

Change in reported number of symptoms before and after ECV was significantly affected by the maintenance of SR at 4 weeks follow-up (assessed with ECG), with patients in SR at follow-up reporting on average fewer AF symptoms than patients that had reverted to AF rhythm (Mean difference = −1.7). The second step included adding both covariates, which did not affect the influence of the successfulness of the ECV on the number of reported symptoms. Engaging in physical activity at baseline was associated with the report of fewer AF symptoms over the assessment period (*p* = 0.01). In a final step, the three emotional distress measures were added. Results showed that the course of depression over the assessed time period was a significant determinant of the change in the reported number of AF symptoms over this period (*p* < 0.0005), meaning that with a larger change in depression score, a larger change in the number of AF symptoms occurred, i.e., for each 1 point increase of the depression score, the reported number of symptoms increased with 0.30 (see Table 6). Anxiety and perceived stress were not significant in this final model, indicating that changes in anxiety and stress were unrelated to changes in the number of AF symptoms.

#### Frequency of symptoms

The reported frequency of AF symptoms was not associated with the time of assessment (before or after ECV; see Table 6 right panel). The change in reported frequency of AF symptoms over time was significantly affected by the maintenance of SR as assessed by ECG-confirmed AF status at follow-up, with patients in SR at follow-up reporting AF symptoms with a lower frequency both before and after ECV than patients that had reverted to AF rhythm (Mean difference = −3.7). Adding both covariates to the model reduced the effect of the successfulness of the ECV to trend level. In the final step we added the measures of emotional distress. Results showed that the course of the reported frequency of AF symptoms was significantly associated with change in depression over this time period, i.e., with 1 point increase in depression score, the AF frequency report also increased 0.75 points (Table 6). Anxiety and perceived stress were not significant in this final model, indicating that changes in anxiety and stress were unrelated to changes in the frequency of AF symptoms.

## Discussion

The objective of the current study was to compare emotional distress levels in AF patients with distress levels in the general population and to examine the relationship between subjective AF symptom reports and emotional distress before and after ECV. Our findings led to two primary conclusions. First, emotional distress levels were substantially higher in the AF group than in the matched sample from the general population and second, higher levels of emotional distress in patients with persistent AF were significantly associated with the report of more AF symptoms during an AF rhythm episode, but also with the change of the report of AF symptoms after ECV. Maintenance of SR after ECV also was an important determinant of the subjective AF symptom report, but to a lesser extent than depression.

Depression was the strongest associate of reporting a larger quantity and a higher frequency of AF symptoms during the AF episode, independent of several covariates, including gender, presence of concomitant cardiovascular disease and physical activity. Our results build on previous research demonstrating negative emotions to be associated with a greater number (Sears et al., 2005) and frequency (Kang, 2006a; Ong et al., 2006) of subjective AF symptoms. Most symptoms of AF originate from the increased heart rate induced by disorganized AF conduction, which may be perceived as chest discomfort. Inadequate blood flow and oxygen delivery may be a consequence of rapid heart rate, and may induce symptoms of fatigue, shortness of breath, light-headedness, edema, and fainting. Nevertheless, the above described symptoms are also perceived by AF patients in the absence of AF rhythm. The depression associated risk for increased report of AF symptoms may in part be explained by the cognitive-affective processing associated with depressive symptomatology, such as rumination and heightened attentional focus on bodily sensation, may influence the report of somatic complaints (Barsky et al., 1988; Howren et al., 2009). Future research may want to focus on methods to reduce the body-centered attentional bias in patients with AF and emotional distress. Frequent asking for symptom reports though, may also have an adverse effect, as patients may become more self-focused because you ask to report about bodily sensations.

Clinically, physicians could screen for depressive or anxious symptomatology to be able to refer highly distressed AF patients to a medical psychologist. Cardiologists should take more time answering questions from anxious patients, which might be accomplished within time constraints by implementing a patient-centered approach of cardiologic care (Jayadevappa and Chhatre, 2011). Psychologists should treat the depression according to prevailing guidelines and additionally focus on reducing the attentional bias toward bodily sensations. There are some techniques that may help, including mindfulness training, which has shown to be able to divert attentional focus (Kee et al., 2012). Moreover, informing patients about the unknown may also alleviate anxiety symptoms as is seen other patient groups (e.g., surgery patients: Bellani, 2008), but still has to be investigated in patients with AF.

Despite initially successful ECV, about 50% of patients had a recurrence at 4 weeks follow-up, which is a normal success rate (Arya et al., 2010). Our results showed significant and stable differences in the level of reported AF symptoms (both number and frequency) between patients who reverted to AF rhythm and patients that had maintained SR, although in the full model, part of this association was explained by differences in depression. We further observed that subjective symptomatic burden of AF did not wane much after ECV in both patient subgroups. In paroxysmal AF it is common that subjective reports of AF symptoms are not in concordance with the real arrhythmic events (Atarashi et al., 2008), and our results suggest that this is also the case in patients with persistent AF. Both maintenance of SR after ECV and change in depression score over the assessment period significantly influenced the course of the reported number and frequency of AF symptoms.

In comparison to the general population, patients with persistent AF experienced higher levels of emotional discomfort, manifested in increased reports of depression, and anxiety, and a higher prevalence of Type D personality. In addition, higher percentages of AF patients scored above the predefined threshold for clinically relevant depression and anxiety. These findings corroborate previous comparisons between patients with other cardiovascular diseases (stroke, hypertension, coronary heart disease) and the general population, showing increased levels of emotional distress across the cardiovascular disease spectrum (Denollet, 2005; Baune et al., 2006). This also confirms several previous reports that showed anxiety levels to be higher in elderly patients with AF as compared to control patients free of cardiac arrhythmia (Thrall et al., 2007; Perret-Guillaume et al., 2010). With regard to depression, studies have reported a higher (Perret-Guillaume et al., 2010) or equal prevalence (Thrall et al., 2007) in AF patients as compared to control patients. The number of patients actually presenting clinically significant levels of anxiety and depression is disturbingly high (between 30 and 50%), and should be a focus point for clinicians and medical psychologists. Clinically, it is thus of importance to address patients’ emotional distress, as a cardioversion might not lead to the expected symptom relief in those patients reporting high levels of emotional distress. Future research should examine whether a similar effect may be observed in patients that are treated with other rhythm therapies, or rate therapy. Moreover, longitudinal studies that employ ambulatory monitoring of AF rhythm should be set up, to examine the role of psychological factors on recurrence of AF episodes in patients with paroxysmal AF episodes. In case of persistent AF episodes terminated by ECV, studies should focus on psychophysiological intermediary processes that may explain ECV success rates, such as sympathetic nervous system activation.

Previous research has shown that there are gender differences in AF prognosis, favoring men with respect to mortality, heart failure, and stroke incidence. Women also tend to be more symptomatic than men, do worse after rhythm control strategies with anti-arrhythmic medication and have a poorer QoL (Dabrowski et al., 2010; Michelena et al., 2010). The current investigation in persistent AF patients however did not find any gender differences in the number of reported AF symptoms as well as the reported frequency with which the AF symptoms occurred, both before and after ECV, a rhythm control intervention. Also, there were no gender differences in the ECG-confirmed AF status 4 weeks after ECV.

Our findings should be viewed in light of several limitations. The attrition rate in our study was unexpectedly high. Approximately half of the included patients were not willing to fill out the second questionnaire, potentially due to the length of the questionnaire which also contained other questionnaires and the short follow-up time of 4 weeks, in spite of the extensive letter explaining the importance of repeated measurements for our research questions. Because of this attrition rate, we cannot be certain that patients who dropped out of the study would have demonstrated the same relations over time, although there were no baseline differences between patients who dropped out of the study and patients that completed the two measurements. A further limitation is our relatively small sample size, which prevented us from including a larger number of covariates. Nevertheless, strengths of our study include the prospective design of the study and the assessment of emotional distress both during AF and after ECV had restored the SR, because this gives insight into the intricate relations between emotional distress, objective AF status and subjective AF symptom reports, and provides ideas for future research. For example, future research could examine whether reducing attentional self-focus affects the number and frequency of reported AF symptoms and whether this affects successfulness of the ECV. Moreover, it should be tested whether emotional distress is a trigger of AF episodes and which psychophysiological processes play a role.

In conclusion, emotional distress is highly prevalent among persistent AF patients and is strongly associated with the number and frequency of reported AF symptoms, independent of gender, concomitant cardiac disease, and cardiovascular risk factors. The subjective symptomatic burden of AF did not wane much after cardioversion, and was significantly determined by the course of depression during this period. These findings call for the attention of clinicians to the high levels of depression and anxiety in this patient population as they are an important determinant of symptom reports, as well as further research on psychological and biomedical factors that may influence the success rate of ECV.

## Conflict of Interest Statement

The authors declare that the research was conducted in the absence of any commercial or financial relationships that could be construed as a potential conflict of interest.

## References

[B1] Arya A. Silberbauer J. S. Vrahimides J. Cheek E. Mitchell A. Boodhoo L. (2010). First time and repeat cardioversion of atrial tachyarrhythmias – a comparison of outcomes. Int. J. Clin. Pract. 64, 1062–1068 10.1111/j.1742-1241.2009.02229.x 20642706

[B2] Atarashi H. Ogawa S. Inoue H. (2008). Relationship between subjective symptoms and trans-telephonic ECG findings in patients with symptomatic paroxysmal atrial fibrillation and flutter. J. Cardiol. 52, 102–110 10.1016/j.jjcc.2008.06.006 18922383

[B3] Barsky A. Goodson J. Lane R. Cleary P. (1988). The amplification of somatic symptoms. Psychosom. Med. 50, 510–519 3186894 10.1097/00006842-198809000-00007

[B4] Baune B. T. Adrian I. Arolt V. Berger K. (2006). Associations between major depression, bipolar disorders, dysthymia and cardiovascular diseases in the general adult population. Psychother. Psychosom. 75, 319–326 10.1159/000093955 16899969

[B5] Beck A. T. Steer R. A. (1993). Manual for the Revised Beck Depression Inventory. San Antonio: Psychological Corporation

[B6] Beck A. T. Steer R. A. Garbin M. C. (1988). Psychometric properties of the Beck depression inventory: twenty-five years of evaluation. Clin. Psychol. Rev. 8, 77–100 10.1016/0272-7358(88)90050-5

[B7] Bellani M. L. (2008). Psychological aspects in day-case surgery. Int. J. Surg. 6(Suppl. 1), S44–46 10.1016/j.ijsu.2008.12.019 19167936

[B8] Camm A. J. Luescher T. F. Serruys P. W. (eds) (2009). The ESC Textbook of Cardiovascular Medicine. New York: Oxford University Press.

[B9] Cohen S. Williamson G. (1988). “Perceived stress in a probability sample of the U.S.,” in The Social Psychology of Health: Claremont Symposium on Applied Social Psychology, eds Spacapan S. Oskamp S. (Newbury Park, CA: Sage), 31–67

[B10] Compare A. Bigi R. Silva P. O. Proietti R. Grossi E. Steptoe A. (2013). Type D personality is associated with the development of stress cardiomyopathy following emotional triggers. Ann. Behav. Med. (in press). 10.1007/s12160-013-9474-x 23494256

[B11] Dabrowski R. Smolis-Bak E. Kowalik I. Kazimierska B. Wojcicka M. Szwed H. (2010). Quality of life and depression in patients with different patterns of atrial fibrillation. Kardiol. Pol. 68, 1133–1139 20967710

[B12] Darbar D. Roden D. M. (2005). Symptomatic burden as an endpoint to evaluate interventions in patients with atrial fibrillation. Heart Rhythm 2, 544–549 10.1016/j.hrthm.2004.11.015 15840484

[B13] Denollet J. (2005). DS14: standard assessment of negative affectivity, social inhibition, and Type D personality. Psychosom. Med. 67, 89–97 10.1097/01.psy.0000149256.81953.49 15673629

[B14] Dorian P. Jung W. Newman D. Paquette M. Wood K. Ayers G. M. (2000). The impairment of health-related quality of life in patients with intermittent atrial fibrillation: implications for the assessment of investigational therapy. J. Am. Coll. Cardiol. 36, 1303–1309 10.1016/S0735-1097(00)00886-X 11028487

[B15] Hagens V. E. Ranchor A. V. Van Sonderen E. Bosker H. A. Kamp O. Tijssen J. G. P. (2004). Effect of rate or rhythm control on quality of life in persistent atrial fibrillation: results from the Rate Control versus Electrical Cardioversion (RACE) study. J. Am. Coll. Cardiol. 43, 241 10.1016/S0735-1097(04)91025-X 14736444

[B16] Howren M. B. Suls J. Martin R. (2009). Depressive symptomatology, rather than neuroticism, predicts inflated physical symptom reports in community-residing women. Psychosom. Med. 71, 951–957 10.1097/PSY.0b013e3181b9b2d7 19779145 PMC2798728

[B17] Jayadevappa R. Chhatre S. (2011). Patient centered care – a conceptual model and review of the state of the art. Open Health Serv. Policy J. 4, 15–25 10.2174/1874924001104010015

[B18] Kang Y. (2006a). Effect of uncertainty on depression in patients with newly diagnosed atrial fibrillation. Prog. Cardiovasc. Nurs. 21, 83–88 10.1111/j.0889-7204.2006.04810.x 16760690

[B19] Kang Y. (2006b). Relation of atrial arrhythmia-related symptoms to health-related quality of life in patients with newly diagnosed atrial fibrillation: a community hospital-based cohort. Heart Lung 35, 170–177 10.1016/j.hrtlng.2006.01.002 16701111

[B20] Kee Y. H. Chatzisarantis N. N. L. Kong P. W. Chow J. Y. Chen L. H. (2012). Mindfulness, movement control, and attentional focus strategies: effects of mindfulness on a postural balance task. J. Sport Exerc. Psychol. 34, 561–579 23027228 10.1123/jsep.34.5.561

[B21] Kupper N. Denollet J. (2007). Type D personality as a prognostic factor in heart disease: assessment and mediating mechanisms. J. Pers. Assess. 89, 265–276 10.1080/00223890701629797 18001227

[B22] Lane D. Carroll D. Ring C. Beevers D. G. Lip G. Y. (2001). Mortality and quality of life 12 months after myocardial infarction: effects of depression and anxiety. Psychosom. Med. 63, 221–230 11292269 10.1097/00006842-200103000-00005

[B23] Lane D. A. Langman C. M. Lip G. Y. H. Nouwen A. (2009). Illness perceptions, affective response, and health-related quality of life in patients with atrial fibrillation. J. Psychosom. Res. 66, 203–210 10.1016/j.jpsychores.2008.10.007 19232232

[B24] Lange H. W. Herrmann-Lingen C. (2007). Depressive symptoms predict recurrence of atrial fibrillation after cardioversion. J. Psychosom. Res. 63, 509–513 10.1016/j.jpsychores.2007.07.010 17980224

[B25] Lavie C. J. Milani R. V. Ventura H. O. (2009). Obesity and cardiovascular disease: risk factor, paradox, and impact of weight loss. J. Am. Coll. Cardiol. 53, 1925–1932 10.1016/j.jacc.2008.12.068 19460605

[B26] Maglio C. Sra J. Paquette M. Dorian P. Bygrave A. Wood K. (1998). Measuring quality of life and symptom severity in patients with atrial fibrillation. Pacing Clin. Electrophysiol. 21, 839

[B27] Michelena H. I. Powell B. D. Brady P. A. Friedman P. A. Ezekowitz M. D. (2010). Gender in atrial fibrillation: ten years later. Gend. Med. 7, 206–217 10.1016/j.genm.2010.06.001 20638626

[B28] Mols F. Pelle A. J. Kupper N. (2009). Normative data of the SF-12 health survey with validation using postmyocardial infarction patients in the Dutch population. Qual. Life Res. 18, 403–414 10.1007/s11136-009-9455-5 19242822

[B29] Ong L. Cribbie R. Harris L. Dorian P. Newman D. Mangat I. (2006). Psychological correlates of quality of life in atrial fibrillation. Qual. Life Res. 15, 1323–1333 10.1007/s11136-006-0029-5 16826433

[B30] Perret-Guillaume C. Briancon S. Wahl D. Guillemin F. Empereur F. (2010). Quality of life in elderly inpatients with atrial fibrillation as compared with controlled subjects. J. Nutr. Health Aging 14, 161–166 10.1007/s12603-009-0188-5 20126966 PMC12878926

[B31] Ploeg van der H. M. Defares P. B. Spielberger C. D. (1980). Handleiding bij de Zelf-Beoordelings Vragenlijst (ZBV). Een nederlandstalige bewerking van de Spielberger State-Trait Anxiety Inventory. [Manual for the ZBV. A Dutch-language adaptation of the Spielberger State-Trait Anxiety Inventory]. Lisse: Swets & Zeitlinger BV.

[B32] Rich M. W. (2009). Epidemiology of atrial fibrillation. J. Interv. Card. Electrophysiol. 25, 3–8 10.1007/s10840-008-9291-5 19160031

[B33] Sears S. F. Serber E. R. Alvarez L. G. Schwartzman D. S. Hoyt R. H. Ujhelyi M. R. (2005). Understanding atrial symptom reports: objective versus subjective predictors. Pacing Clin. Electrophysiol. 28, 801–807 10.1111/j.1540-8159.2005.00171.x 16105008

[B34] Smith D. Lip G. Y. Lane D. A. (2010). Impact of symptom control on health-related quality of life in atrial fibrillation patients: the psychologist’s viewpoint. Europace 12, 608–610 10.1093/europace/euq083 20353966

[B35] Spielberger C. D. (1983). Manual for the State-Trait Anxiety Inventory. Palo Alto, CA: Consulting Psychologists Press Inc.

[B36] Thrall G. Lane D. Carroll D. Lip G. Y. H. (2006). Quality of life in patients with atrial fibrillation: a systematic review. Am. J. Med. 119, 448.e1–19. 10.1016/j.amjmed.2005.10.057 16651058

[B37] Thrall G. Lip G. Y. Carroll D. Lane D. (2007). Depression, anxiety, and quality of life in patients with atrial fibrillation. Chest 132, 1259–1264 10.1378/chest.07-0036 17646231

[B38] van den Berg M. P. Ranchor A. V. Van Sonderen F. L. Van Gelder I. C. Van Veldhuisen D. J. (2005). Paroxysmal atrial fibrillation, quality of life and neuroticism. Neth. J. Med. 63, 170–174 15952485

[B39] Volgman A. S. Manankil M. F. Mookherjee D. Trohman R. G. (2009). Women with atrial fibrillation: greater risk, less attention. Gend. Med. 6, 419–432 10.1016/j.genm.2009.09.008 19850238

